# Decolonizing Animal Welfare Through a Social Justice Framework

**DOI:** 10.3389/fvets.2021.787555

**Published:** 2022-01-27

**Authors:** Johnny L. Jenkins, Mueni L. Rudd

**Affiliations:** Department of Research and Development, Companions and Animals for Reform and Equity (CARE), Baltimore, MD, United States

**Keywords:** social justice, human-animal bond (HAB), animal welfare, disability justice, environmentalism, gender and sexuality diversity, racial equity

## Introduction

A shift in animal welfare approaches has centered more attention on the human-animal bond (HAB) between diverse people and other animals (1). Scientific curiosity into the significance of these bonds is led by prestigious academic research institutions and organizations like the Human-Animal Bond Research Institute (HABRI). Although interdisciplinary study into the phenomena has become prevalent, animal bond disparities between historically excluded populations persist. Companions and Animals for Reform and Equity (CARE) argue for social scientists and animal welfare researchers to probe further into the human-animal relationship critically through race, ethnicity, class, sex, and gender frameworks to reimagine a welcoming and inclusive sector for marginalized communities.

Historically, police brutality within communities of color has been broadly documented. George Floyd's death in May 2020 served as a catalyst that ignited a global call and response for racial equity and justice. Likewise, animal welfare in the United States has responded with countless diversity, equity, and inclusion centered discussions and initiatives. Earlier in 2020 CARE, the country's first Black-led animal welfare organization was founded to prioritize inclusion as a key tool in lifesaving and human and animal well-being. Community participatory research and personal storytelling are key to examining the impact of disparities in animal welfare. Two challenges persist: Black, Indigenous, and other people of color (BIPOC) remain noticeably absent, while perceptions of tokenism, colorism, and texturism slant the national dialogue toward white fragility as opposed to BIPOC-centered solutions to increase inclusion within the sector. Nevertheless, these conversations continue as an essential discourse to healing the national racial divide across all sectors, including animal welfare.

CARE focuses on comprehensive human and animal well-being through the pursuit of community wisdom that will drive lifesaving activities in marginalized communities. Doing so requires adopting transformative justice principles into a human and animal well-being framework. As a result, community engagement strategies and programs will result impacted by more welcoming, culturally competent, and responsive spaces for BIPOC communities in animal welfare. Animal welfare must expand the narrow notion of well-being to include critical justice issues like gender and sexual diversity, racial equity, economic and housing security, disability rights, and environmentalism.

## Disability Justice

Disability justice may appear to be a notable outlier in much of justice advocacy and studies. In accessibility can easily be described as one of the most impactful barriers to disability justice. Divesting from that requires intellectually and physically disabled populations to be visible and centered in the movement toward inclusive social justice. Scholar advocates like Radical Disabled Women of Color United (2) urge for a critical intersectional disability studies approach that prioritizes the meeting of race, gender, class, and disabilities. Disabled lived experiences with systems of oppression essentially provide increased validity to justice advocacy frameworks across interdisciplinary and cross-sector approaches.

Animal welfare to outwardly appears to be in agreement that the utilization of service animals is necessary. Despite this, the fight for emotional support service animals to be legitimized continues for people living with a disability and requiring this form of support. Current federal legislation does not define emotional support service animal training as work or a task for the animal ignoring the direct feedback from disabled communities (3). Legislation supporting communities with visual impairments and their service animals reveals bias and lack of disabled community involvement in policy creation. This is merely one example of the lack of commitment to cross-disability solidarity at the intersection of disability justice and animal well-being (4).

Ableism is as normative to our society as colonial whiteness. Reimagining disability justice for animal and human well-being challenges advocates like CARE to create a culture of access in the outputs to the community. Disability justice advocacy is most simply placing value on the advancement of equitable access practices despite the lack of societal buy-in from individuals, institutions, and government.

## Environmental Justice

Climate experts like Dr. Ayana Johnson (5) acknowledge that the environmental justice movement improves by being interconnected to race and class liberation work. This requires environmental justice support and solutions to be centered around minoritized communities in order to be sustainable models. If we think of environmental racism as an extension of harmful state sanctioned practices then we can more effectively theorize it as a form of state violence. The largely white western environmental movement has not prioritized racially diverse communities. BIPOC populations are disproportionately exposed to environmental racism like proximity to highways landfills, chemical facilities, or toxic waste. The associated health risks like cancer or asthma impact the humans and other animals in those communities. Animal advocates must consider combining efforts with related human service organizations to improve services to all.

Environmental Scholar, Dr. Pellow (6) continues on environmental justice with critical environmental justice studies, which seek to expand the field of environmental justice to move beyond its conceptual, theoretical, and methodological limitations. Their approach draws from numerous fields of scholarship to produce more robust arguments resulting from the examination of the persistent occurrence of environmental injustices that impact humans and non-humans. Notably, the approach offers animal welfare an opportunity to reimagine old paradigms through intersectional theory to define new strategies for BIPOC community engagement and coalition-building. The promise of critical environmental justice studies lies in its capacity to explain the sources and consequences of our socio-ecological crises and develop more generative analyses of how social change efforts within and across species may meet those challenges more fully.

Issues of environmental racism like communities of color and their animals' discorporate exposure to health risks, food deserts, and proximity to toxic facilities or environmental hazards can all be more easily understood using environmental justice frameworks to reimagine deliberate support. It is not the intent of the authors to appear anthropocentric in presented views of human-animal relationships. Instead, the desire is to improve what Critical Animal Studies hopes to do by centering the needs and lived experiences of historically oppressed humans who have companion animals for the purpose of keeping those relationships sufficiently supported.

## Gender and Sexual Diversity Justice

Although animal welfare has embraced research that examines the human-animal bond (1), the sector has only scratched the surface to use the knowledge to address disparities and inequities experienced by LGBTQ+ communities. Further extension into sexualized communities is likely to elucidate even more fertile research and program designs that cater to their unique challenges. In order to engage such challenges, animal welfare researchers must embrace immersive critical research rigor practice that fearlessly intersects animal welfare with race, sexuality, and gender. Walsh (7) underscores how transgender, non-binary, and older sexual minorities who experience social stigma value the nonjudgmental acceptance of companion pets (8). Additional research from the National Health, Aging and Sexuality Study (NHAS) demonstrates that sexual and gender minorities in midlife and later life are at elevated risk for disability, poor physical health, and depression (9). Nevertheless, uncharted opportunities remain elusive to designing innovative, solution-oriented community-based programs to address isolation among aging sexual minorities.

## Racial Justice

Critical race scholars suggest that harm reduction and equitable societal progression surrounding race cannot exist without acknowledgment of racialized lived experiences (10). Racial justice studies promote frameworks that value understanding the differences in institutional, structural, interpersonal, and internalized racism to effectively reimagine equity efforts for non-white populations' quality of life (11). It is vital to humanize these experiences in racial justice work across sectors by recognizing racial trauma's impact on the individual and their community. A race-based traumatic impact can present itself as increased aggression, vigilance/suspicion, sensitivity to threats, psychological/physiological symptoms, alcohol/drug usage, or a narrowing sense of time (11). Harper (10) does clarify that critical race theory does not need to work alone in reimagining equitable approaches. Therefore, there is a healthy argument for the intersectional relationship race and other systems of oppressions mentioned in this article have on building equitable frameworks of justice-based support for humans and their animals. The racially diverse individuals and families served by animal welfare require sensitivity and trust-building components that honor their lived experiences. Additional modifications to practices are required for racially diverse individuals who also experience intersections of poverty or economic hardships and gender differences.

The CARE/Harvard Project Implicit study (12) identified significant negative attitudes toward racially diverse, specifically Black, and poor, animal owners within the animal welfare sector. In addition, the study collected demographic data that supports the casual narrative that animal welfare is predominantly white-run and operated. This disparate representation in the field dictates the types of services and approaches prioritized that lack centrality in equity or justice to establish animal welfare as welcoming to BIPOC communities. The study also revealed significant differences in attitudes and perceptions toward various racial groups that suggest a racial hierarchy. Although non-white communities in animal welfare are all subjected to harmful practices there are differences in treatment between people of color by white authority and leadership in animal welfare. Likewise, Crenshaw (13) argues that the surface level grouping of white and non-white can be harmful due to the very different racialized lived experiences. Reimagining and co-creating racial justice for human and animal well-being requires divesting from the current monolithic perspective that prevails. Animal welfare has an opportunity to evolve by acknowledging the intersectional realities of BIPOC lived experiences and to support comprehensive human and animal well-being ethically and respectfully.

## Discussion

Little research is available to provide an explanation as to why animal welfare scholars, advocates, practitioners, and grant makers maintain such minuscule data specific to Black, Indigenous, and other people of color. The University of Michigan Health recently published a survey study to better understand the needs for companion animal care for individuals requiring hospitalization. Despite the study's ability to generate discussion on access to emergency boarding and companion animal fostering there appears to be a lack of prioritization for racial diversity by the researchers. Polick et al. (14) report “Race/ethnicity was initially categorical but, due to the low frequency of responses in seven non-white categories, this item was dichotomized.” This is harmful to racially diverse communities that own companion animals for a few reasons. The researchers bulk all BIPOC individuals into one as a convenience that inherently tells the animal welfare sector that experiences are either white or non-white. This is seen as an erasure of complex racial experiences because all non-white experiences are not the same. Additionally, the authors state 62% of the sample pool used identified as white. When animal welfare references published articles like the mentioned study to support programing they are prioritizing white experiences and needs to inform their work. Monolithic approaches to reporting racial diversity, lack of clear race or ethnic breakdowns, and significantly small sample pools of people of color contribute to harm in our field yet continue to occur.

A query that explores the historical impact of the concept of race neutrality within animal welfare would be worthy of examination. Floyd's death elucidated the need for more scholarly research and grassroots data collection within the academy, national animal welfare organizations, veterinary practices and animal grant makers. Additional research that provides an extensive analysis of the human-animal bond between intersectional communities would benefit community-based programmatic design and outreach.

Once this research is inevitably pursued, researchers should practice due diligence to ethically implement research methodologies that partner with marginalized communities as participants as opposed to subjects. Participatory action research methodologies are available that follow a social justice framework that mitigates the historically harmful, and sometimes violent nature, in which knowledge has been unethically extracted from marginalized communities (15).

We argue animal welfare must build authentic relationships with intersectional BIPOC communities to holistically address the challenges that impact these communities and their pets. In essence, this work requires the disruption of the status quo within animal welfare to benefit pets within marginalized communities.

## Author Contributions

All authors listed have made a substantial, direct, and intellectual contribution to the work and approved it for publication.

## Funding

The opinion article submitted by the Department of Research and Development is solely supported by Companions and Animals for Equity and Reform (CARE).

## Conflict of Interest

The authors declare that the research was conducted in the absence of any commercial or financial relationships that could be construed as a potential conflict of interest.

## Publisher's Note

All claims expressed in this article are solely those of the authors and do not necessarily represent those of their affiliated organizations, or those of the publisher, the editors and the reviewers. Any product that may be evaluated in this article, or claim that may be made by its manufacturer, is not guaranteed or endorsed by the publisher.
